# Antimicrobial stewardship in veterinary medicine: a review of online resources

**DOI:** 10.1093/jacamr/dlad058

**Published:** 2023-05-26

**Authors:** Fergus Allerton, James Russell

**Affiliations:** Willows Veterinary Centre & Referral Service, Highlands Road, Shirley, Solihull, West Midlands, B90 4NH, UK; Airdrie, Oldfields Road, Uttoxeter, ST1 48DE, UK

## Abstract

**Background:**

An awareness of antimicrobial resistance and stewardship is important for all prescribers from a One-Health perspective. Educational resources have been created to help veterinary practitioners and encourage an optimized approach to their antimicrobial use.

**Objectives:**

To support veterinarians to select the most appropriate educational resource to meet their personal learning goals in relation to veterinary antimicrobial stewardship (AMS).

**Methods:**

Modular online platforms, developed to promote AMS in veterinary practice (farm and companion animal), were reviewed and key features highlighted, including the required time commitment, resource type, focus and source as well as a subjective evaluation of resource accessibility (according to prior knowledge base).

**Results:**

Five different online courses are described in this educational resource review: Antimicrobial stewardship in veterinary practice; Farm Vet Champions; Farmed Animal Antimicrobial Stewardship Initiative (FAAST); the Pathway of antimicrobial resistance (AMR) for a veterinary services professional; and the VetAMS online learning program. Each of these tools introduces users to key themes of veterinary AMS. Upon completion of any of the courses practitioners should feel confident to assume an active role as proponents of rational antimicrobial use. Significant differences between resources, relating to the focus (companion or farm animal), scope and depth of material covered, are recognized reflecting their respective target audiences.

**Conclusions:**

Several informative and accessible resources, centred on the core principles of veterinary AMS, were reviewed. Key features have been highlighted to inform and guide resource-users towards the most appropriate tool for them. Greater engagement with these educational resources will hopefully contribute to improved antimicrobial prescribing among veterinarians and greater awareness of the importance of stewardship for the profession.

## Introduction

The significant impact on human health of antimicrobial resistance (AMR) is widely documented,^1,2^ with global mortality attributable to MDR infections anticipated to continue to grow.^3^ Quantification of the direct consequences of AMR on animal health is lacking. Nonetheless, the negative implications for animal morbidity, mortality and reduced productivity are well recognized within the veterinary sector^4–7^ emphasizing the need for coordinated efforts to address this emerging phenomenon.^8^

MDR bacteria have been isolated from farm^9^ and companion^10^ animals. Zoonotic transmission of resistant strains of bacteria or interbacterial exchange of the genetic material conferring resistance, have also been recorded from pets and livestock.^11–13^ Given the overlap of antimicrobial classes between human and veterinary medicine, antimicrobial use in animals could amplify levels of AMR among pathogens of public health concern.^14,15^ In recognition of this, potentially vast, reservoir of AMR, veterinarians are considered critical stakeholders in the One Health approach to antimicrobial stewardship (AMS).^16^ The One Health concept recognizes the interdependent needs of people, animals and the environment; an integrated approach is required to balance and optimize the health of each element.

Current awareness of AMR and rational antimicrobial use guidance was generally poor among surveyed final-year veterinary students in Europe,^17^ Australia^18^ and Nigeria^19^ highlighting a need for further training in this area. The proportion of veterinarians working in companion animal, equine, farm or mixed practice varies considerably according to geographical location.^20–22^ Therefore, educational resources must be designed to meet the needs of a diverse diaspora of veterinarians. They should provide up-to-date, evidence-based information succinctly to a time-poor community. Different tools have adopted a variety of approaches to achieve these aims.

Educational resources have an important role to inform veterinarians and encourage optimized antimicrobial prescribing habits. The objective of this educational resource review was to look at available modular programmes that promote AMS in veterinary practice and to inform potential users of their relative merits. AMS describes a coherent set of actions that promote the responsible use of antimicrobials (the right drug, at the right time, with the right dose, for the right duration and via the right route). The educational resources reviewed here offer an introduction into this important topic for veterinary practitioners.

## Materials and methods

We included modular, freely accessible, online veterinary AMS educational resources that had been previously reviewed in *JAC-AMR* or that were known to the authors. The educational resources listed in Table 1 were selected for this educational resource review.

**Table 1.** Resources included in this educational resource review

Educational resource	Course provider	Link
Antimicrobial stewardship in veterinary practice	British Society for Antimicrobial Chemotherapy	https://www.futurelearn.com/courses/antimicrobial-stewardship-in-veterinary-practice
Farm Vet Champions	RCVS Knowledge	https://learn.rcvsknowledge.org/course/index.php?categoryid=6
Farmed Animal Antimicrobial Stewardship Initiative (FAAST)	Ontario Veterinary Medical Association	https://www.amstewardship.ca/
Pathway of AMR for a veterinary services professional	The Fleming Fund and Open University	https://www.open.edu/openlearncreate/course/view.php?id=5352
VetAMS online learning program	AMR Vet collective	https://www.vetams.org/

RCVS, Royal College of Veterinary Surgeons.

Each resource was accessed and reviewed independently by both authors. Information was recorded about each resource, including the anticipated duration; whether there was any associated accreditation or certification; available language options; whether or not the resource is reusable/downloadable; cost of access; area(s) of focus of the resource; and the target audience including relevance and applicability of the resource for practitioners working in different regions of the world.

Further information collated for each resource included the multimedia resources used for learning (narrated video, quizzes, case studies) and key messages expressed. We have also outlined where different resources may complement each other or cover similar ground. The anticipated level of base AMR knowledge (basic, intermediate and advanced) appropriate for each education resource has been subjectively assessed, using the authors’ judgement, to offer an indication of the target audience.

Particular features of some resources have been highlighted to offer a more detailed insight where appropriate.

## Results

The massive open online course, or MOOC (*Antimicrobial stewardship in veterinary practice*), from the British Society for Antimicrobial Chemotherapy (BSAC) is most appropriate for veterinarians with little prior knowledge and experience of AMR. The information presented in each section offers a brief introduction to the topic, whereas web links to additional material allow interested readers to delve deeper into specific areas. The bite-size units are coherently grouped by key themes, including an overview of AMS; the mechanisms and development of AMR; barriers to implementation of AMS; monitoring and benchmarking antimicrobial use; and strategies to communicate the AMS message to clients.

**Figure 1.**
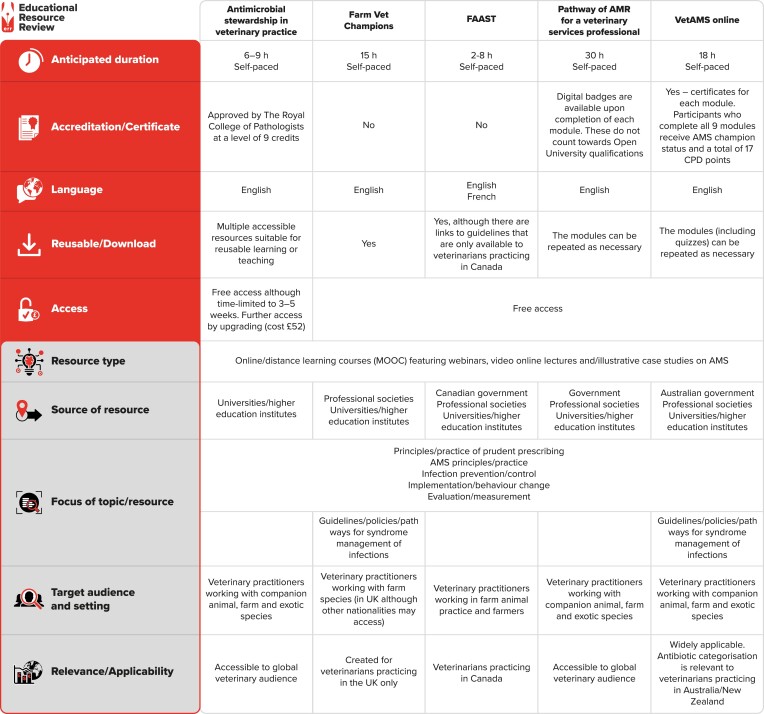
Features of the educational resources. CPD, continuing professional development.

Completion of the BSCAC MOOC is recognized by the Royal College of Pathologists (Figure 1). A certificate of achievement is also available to users who pay for extended access to the FutureLearn^®^ platform. For all learners, tangible objectives to achieve upon completion of each week’s course are outlined. Even where case studies may be focused on unfamiliar species, the underlying principles are applicable across all veterinary settings.

The *Farm Vet Champions* and *FAAST* platforms are tailored towards farm animal practitioners, with both offering dedicated modules relating to specific sectors of the agricultural industry (e.g. poultry, sheep, pig, beef and even aquaculture). The FAAST module on AMR begins with a short (5 min) animated film providing an overview of AMS and the ‘5 Rs’ (responsibility, reduction, refinement, replacement and review). This subject is later expanded upon in a more detailed 15 min film highlighting the human and animal health risks from AMR and the importance of good stewardship practice. Background information is also provided to support veterinarians when having conversations with their clients. Potential barriers to rational antimicrobial use are recognized, and links provided to guidelines for multiple species (only accessible to veterinarians in Canada) as well as example tools (e.g., treatment algorithms for calf neonatal diarrhoea). This introductory module will mainly be useful for veterinarians working in farm animal practice who are new to the subject of AMS. The other 13 sections in this tool are aimed primarily at livestock producers and are sector specific. They contain few multimedia resources but offer examples of ways to implement the 5 Rs in practice.

*Farm vet champions* has been specifically designed for veterinarians working in the UK; non-UK residents are asked to contact the site administrator to gain access to the modules. The section on legal and responsible use of medicines may include national particularities that do not apply in other countries. Nonetheless, the majority of the presented information and stewardship recommendations are applicable everywhere, and the platform uses a variety of modalities, including podcasts, videos and animations, to convey the material in a clear and engaging manner. The platform also encourages users to set up Specific Measurable Achievable Realistic and Time-bound (SMART) goals that can bring their learning to the fore. These goals can be set individually or across teams by invitation. Furthermore, there are links to other providers that offer a central platform to collate, report and compare antimicrobial use for cattle, sheep, pigs or poultry.

*Tackling antimicrobial resistance*, a MOOC developed by The Fleming Fund and The Open University, comprises a series of 25 online modules divided into different pathways suitable for laboratory, clinical and veterinary professionals and policy makers. The veterinary track contains several modules that are not specifically orientated towards a veterinary audience. Whereas all modules offer valuable learning opportunities, those that reference animal health may offer more directed learning and tangible benefit for veterinarians. Each module begins with a short assessment to test the reader’s knowledge and clarify learning outcomes. Practical real-world case studies are presented, covering a broad range of species from livestock to fish. Readers are invited to prepare their own thoughts and then check them against a model answer. A mark of >50% is required on completion of a module quiz to receive a digital badge.

The *VetAMS online learning program*, developed by contributors from Australia and New Zealand, comprises nine modules including core concepts in AMR, antibiotic classes, mechanisms of resistance, infection control and using prescribing guidelines. This platform is most suited for practitioners working with companion animals. Each module offers a variety of learning styles (recorded webinars, podcasts, animations and quizzes) to support participants. The final module focuses on practical tips to implement a stewardship programme. Additional links are provided to freely accessible tools including a downloadable, prescribing practices poster and the Australian Veterinary Association guidelines for prescribing, authorizing and dispensing veterinary medicines (applicable to livestock and companion animals). This course is hosted on the AMR Vet Collective webpage, a site that includes a regularly updated blog page with topical stories designed to bring AMR material to a wider veterinary audience and links to current peer-reviewed guidelines relevant to veterinarians working in Australia and New Zealand.

**Figure 2.**
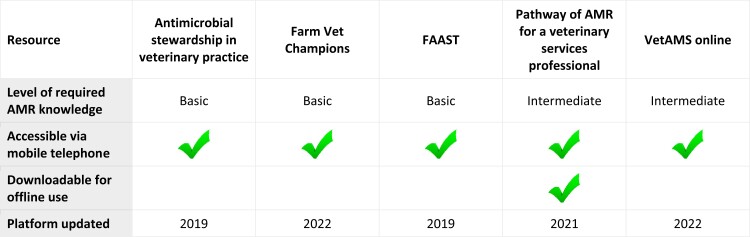
Further features of the educational resources.

Fundamental terminology is systematically explained in all resources with pertinent examples allowing participants to follow without additional research. Modules contained within all the resources provide a mechanistic explanation of antimicrobial action and bacterial resistance. There are, however, differences in the scope and depth of material covered across these resources (Figure 2) and readers may find some of the material presented by the Fleming Fund more technically complicated. The modular designs enable participants to select training of greatest relevance to their roles and preferred learning style.

## Discussion

These five educational resources, all MOOCs, offer participants an introduction to key themes of veterinary AMS. Upon completion of any of the courses practitioners should feel confident to assume an active role as proponents of rational antimicrobial use. The skills acquired can also be shared with colleagues to ensure a trickle-down effect of this information. The role of education to improve AMS has been repeatedly advocated in veterinary literature.^23–27^ Empowering veterinarians (and farmers) with an improved awareness of the currently available evidence should guide improvements in stewardship across different livestock sectors. Knowledge gaps in this field have been identified among farmers in Bangladesh,^28^ the UK^29^ and the USA,^30^ and crucially among veterinarians working in India,^31^ the Netherlands,^32^ Nigeria^33^ and the USA.^34^ AMS is undoubtedly a global phenomenon; these open-access resources provide an opportunity to try to redress any geographical knowledge gaps.

Online educational resources have previously been developed for medical healthcare professionals including a MOOC designed by the University of Dundee and the BSAC to support AMS in low- and middle-income countries. The utility of that resource to fulfil a global learning need was analysed to help future resource-creators to optimize the difficulty level, usefulness and time taken to complete each week’s programme.^35^ Overall, qualitative feedback was very positive and participants felt empowered to translate their new knowledge and skills into action.^35^ This MOOC served as a model for the subsequent veterinary version, which has similar aims to fulfil a global knowledge gap.

Impact assessments have not been published for the educational resources reviewed in this study although the need for evidence-based validation of other forms of stewardship tool has been recognized in veterinary medicine.^36–38^ For example, the quality and implementability of Australian guidelines for antimicrobial prophylaxis for surgery on dogs and cats has been assessed using established evaluation tools—the GuideLine Implementability Appraisal (GLIA) and the Appraisal of Guidelines for Research and Evaluation version 2 (AGREE II)^36^—and the impact of the Swiss online antibiotic use guidance tool (AntibioticScout.ch) on antimicrobial prescriptions in cats has been calculated.^37^ Further research is indicated to measure the influence of educational resources on prescribing behaviours of veterinarians, to establish a tangible benefit of their use.

Optimal learning strategies vary by individual and career stage and encompass both active and passive learning pedagogies.^39^ Improved appreciation and knowledge of AMR was detected among final-year medical students in Brazil who followed an online course with a mix of learning methods, supporting the use of this teaching modality.^40^ Opportunities to improve educational resources provided for human healthcare workers have focused on increasing engagement with students through active learning sessions that allow the students to integrate knowledge with practice.^41,42^ Provision of active learning is inherently more challenging via a remote platform; however, individual challenges and end-of-module quizzes are included in these resources to facilitate user engagement and interest. These complement the passive learning offered via narrated videos, animations and podcasts. These didactic offerings are particularly important to convey a theoretical understanding of AMR and the decision-making processes that underlie rational antimicrobial selection.

The importance of communication as a stewardship facilitator has been emphasized in studies in medical primary care,^43^ and communication skill training contributed to reduced prescription rates in children.^44^ Successful implementation of any antimicrobial use policy hinges on the active engagement of multiple different parties. Educational resources offer a gateway to specific techniques that support bringing about sustained behavioural change among prescribers—the key objective of stewardship interventions.^45^ Several resources (Farm Vet Champions, FAAST and BSAC) offer sections dedicated to the development of effective communication skills. Practical measures are proposed that could help improve the outcome of stewardship-centric conversations with strategic stakeholders (e.g. farmers or pet owners).

In human hospitals, educational interventions are frequently presented with concomitant stewardship interventions such as prospective audit and feedback.^46^ This was similarly demonstrated in these reviewed resources. For participants seeking actionable measures that can be introduced within their own practice, there are additional tools in association within some of these resources that support practitioners to turn their learning into action and help them manage their antimicrobial use in practice. The role of a farm vet champion, similar to the premise of an antibiotic guardian, emboldens veterinarians to select personal SMART goals and assume ownership of their prescribing practices. The act of making such pledges can serve as a motivating force^47^ to reinforce the learnt material.

Inevitably, there will be some gaps in any educational resource, and participants may gain more from selected sources according to their prior knowledge of the subject and their learning styles. The remote accessibility provides unlimited flexibility but compromises the capacity to network with like-minded individuals. The use of regularly updated and moderated community forums or discussion boards could help address this problem but are not a feature of any of these resources. The resources have all been produced in high-income countries and demand a base level of facilities, which may not be available in low- and middle-income nations.

Veterinary AMS is a critical component of the wider One Health movement aiming to raise awareness of AMR among antimicrobial prescribers. Each of the educational resources reviewed here provides users with an understanding of key AMS themes, including the mechanisms of AMR and the importance of rational antimicrobial use, and offers strategies to communicate this information to pet owners and farmers. These resources evoke a variety of learning styles and cover different, sector-specific content all with similar objectives. Future research should look to evaluate the success of these resources in achieving meaningful behavioural change among users and to determine their impact on antimicrobial prescription.
